# Malaria surveillance and case management in remote and indigenous communities of Panama: results from a community-based health worker pilot

**DOI:** 10.1186/s12936-022-04318-z

**Published:** 2022-10-21

**Authors:** Darlene Bhavnani, Bernardo García Espinosa, Madeline Baird, Nicholas Presley, Arnaud Le Menach, Christina Bradley, Marcela Outten, Oscar González

**Affiliations:** 1 Clinton Health Access Initiative, Panama City, Panama; 2 Ministerio de Salud de Panama, Panama City, Panama; 3 Department of Population Health, Dell Medical School, University of Texas at Austin, Austin, Texas United States

**Keywords:** Malaria, Community health worker, Rapid diagnostic test, Surveillance, Case management, *Plasmodium vivax*, Guna Yala, Elimination

## Abstract

**Background:**

Panama is one of eight countries in Mesoamerica that aims to eliminate malaria by 2022. Malaria is concentrated in indigenous and remote regions like Guna Yala, a politically autonomous region where access to health services is limited and cases are predominately detected through intermittent active surveillance. To improve routine access to care, a joint effort was made by Guna Yala authorities and the Ministry of Health to pilot a network of community health workers (CHWs) equipped with rapid diagnostic tests and treatment. The impact of this pilot is described.

**Methods:**

Access to care was measured using the proportion of villages targeted by the effort with active CHWs. Epidemiological impact was evaluated through standard surveillance and case management measures. Tests for differences in proportions or rates were used to compare measures prior to (October 2014-September 2016) and during the pilot (October 2016-September 2018).

**Results:**

An active CHW was placed in 39 (95%) of 41 target communities. During the pilot, CHWs detected 61% of all reported cases from the region. Test positivity in the population tested by CHWs (22%) was higher than in those tested through active surveillance, both before (3.8%) and during the pilot (2.9%). From the pre-pilot to the pilot period, annual blood examination rates decreased (9.8 per 100 vs. 8.0 per 100), test positivity increased (4.2% to 8.5%, Χ^2^ = 126.3, *p* < 0.001) and reported incidence increased (4.1 cases per 1000 to 6.9 cases per 1000 [Incidence Rate Ratio = 1.83, 95% CI 1.52, 2.21]). The percent of cases tested on the day of symptom onset increased from 8 to 27% and those treated on the day of their test increased from 26 to 84%.

**Conclusions:**

The CHW network allowed for replacement of routine active surveillance with strong passive case detection leading to more targeted and timely testing and treatment. The higher test positivity among those tested by CHWs compared to active surveillance suggests that they detected cases in a high-risk population that had not previously benefited from access to diagnosis and treatment. Surveillance data acquired through this CHW network can be used to better target active case detection to populations at highest risk.

## Background

Malaria is a significant cause of global morbidity and mortality. In 2020, there were an estimated 241 million cases of malaria and 627,000 deaths. The region of the Americas made up less than 1% of this global burden [1]. To date, three countries from the region have been granted elimination status by the World Health Organization [1]. Driven by the global momentum for eradication and significant progress made across the Americas, eight countries in Mesoamerica are participating in a regional initiative to eliminate malaria by 2022. However, progress has been uneven across the region. In 2020, Costa Rica, Nicaragua and Panama, reported an increase in malaria incidence relative to 2015; The number of locally-acquired cases reported in Panama increased fourfold, as it rose from 546 in 2015 to 2190 in 2020 [1]. Approximately 99% of cases reported in Panama are caused by *Plasmodium vivax* [2].

Malaria burden in Panama is concentrated in indigenous, remote and otherwise hard to reach populations with limited access to health services. In 2018, 98% of all locally acquired cases in Panama originated in one of four endemic areas (Ngäbe-Buglé, Darién, Panamá Este, and Guna Yala), with 42% of cases reported from the Guna Yala alone [2]. Guna Yala stretches along the Northeastern coast of Panama and is geographically isolated from the rest of the country. While malaria care at public health facilities is provided at no cost, poor access due to geography and climate, and the cultural ties to traditional medicine in Guna populations, limit treatment-seeking behaviour for fever at these facilities [3–5].

Panama has long since recognized the challenges to malaria surveillance and case management in remote and indigenous areas like Guna Yala. The Department of Vector Control within the Ministry of Health (Ministerio de Salud de la República de Panamá, MINSA) has relied heavily on a cadre of vector control technicians (VCTs) to control malaria through active surveillance [6]. Active surveillance is targeted to endemic communities approximately every two weeks; VCTs visit each house in the community, screening household members for current fever, or fever within the last 30 days with intermittent chills and sweating. Thick blood smears are taken from those with fever and transported to a central laboratory in Guna Yala where they are read. Cases are reported through a standardized case reporting form and treatment is administered through directly observed therapy. Additional efforts made by VCTs include case investigation, reactive case detection, indoor residual spraying and thermal fogging as well as targeted mass drug administration to control outbreaks [7, 8].

Despite the large investment and high level of effort made by the Department of Vector Control to visit endemic communities routinely, their intermittent presence in remote communities has resulted in inconsistent access to diagnosis and treatment. In practice, VCTs confront logistical challenges traveling to communities, including resource constraints and extreme weather conditions that can make active surveillance visits at the desired cadence nearly impossible. Communication is also limited in the area, hindering the department’s ability to negotiate their activities with local leaders ahead of time. When active surveillance visits are made, highly mobile populations that reside in the community and those not presenting with symptoms may be missed. Additionally, Panama’s prior reliance on microscopy for diagnosis required lengthy travel to a single laboratory in Guna Yala, resulting in delays in confirmation and notification of up to one month or more [6, 7]. Individuals with fever living in high-risk areas were sometimes treated and investigated presumptively [7]. Furthermore, while directly observed therapy has been shown to improve treatment outcomes in persons infected with *P. vivax* [9], it is resource intensive and has not been systematically implemented given the department’s engagement in other malaria activities.

An assessment completed in 2015 identified the need for additional complementary case management and surveillance strategies to improve access to health services in communities at risk for malaria [10]. Complementary strategies included the introduction of community-based service delivery through a community health worker (CHW) network. CHWs equipped with malaria rapid diagnostic tests (RDTs) can help to extend health services to remote populations by providing patients with consistent access to diagnosis and treatment in their communities [11]. Moreover, use of malaria RDTs presents potential for alignment with the World Health Organization’s (WHO) recommendation that results of a parasitological diagnosis be available within two hours of a patient presenting to a health worker [12]. Together with Guna governance, MINSA and Clinton Health Access Initiative (CHAI) piloted a CHW network and use of RDTs to extend timely diagnosis, treatment and surveillance to communities in Guna Yala. This programme evaluation aims to: (1) Describe improvements in access to care; (2) Assess the quality of the care delivered by CHWs and; (3) Evaluate the pilot’s impact on the epidemiology of malaria in Guna Yala.

## Methods

### Study site and population

Guna Yala includes approximately 480 km of Caribbean coastline that extends Southward to Colombia [13]. Its population is projected to be approximately 46 K [14]. Most residents live in low-lying coastal areas on the mainland and nearby islands [15]. The region is accessible by one main road, sea and air but both heavy rain and strong wind can limit mobility; heavy rain typically falls between May and December [3].

Guna Yala has distinct cultural and economic practices compared to the rest of Panama. The Guna people practice traditional medicine and conceptualize illness through a lens of spirituality [4]. Guna Yala’s Multidimensional Poverty Index (MPI), an indicator of education, health and standard of living in children younger than 18 years [6], was four times below the national average in 2018 [16]. Life expectancy, literacy rates and housing conditions also fall behind the rest of the country. Mortality in children younger than one year is twice as high in Guna Yala compared to the national average [16, 17].

Guna Yala was selected as the study site for the pilot given the high incidence of malaria and the level of interest expressed by its leadership. Guna Yala is governed by a General Guna Congress led by three “Caciques” and composed of political and spiritual community leaders called “Sailas”. The Guna Congress recognizes 51 communities in the *comarca* that fall within one of four larger political subdivisions called *corregimientos*. All communities are ethnically Guna, apart from two communities in the corregimiento of Puerto Obaldía, which identify culturally as Latino [18]. Puerto Obaldía borders Colombia and is one of the main crossing points for migrants heading north [19].

### Community health worker network launch

In September 2016, a proposal was approved by the Guna Yala Congress to initiate a two-year CHW pilot. One CHW per target community would be trained to conduct malaria RDTs and to provide treatment to those testing positive. This pilot would be layered on top of existing public health activities, including active surveillance conducted by VCTs.

Communities were targeted for the intervention using reported case burden in the past three years, population size, malaria risk [20] and access to health facilities. The list of targeted communities (ranging in size from approximately 100–2000 persons) was evaluated and refined approximately every six months during the two-year pilot. On average, a CHW covered about 600 people. The local Department of Vector Control worked with *Sailas* to identify and recruit respected community members according to predetermined criteria, including Spanish literacy, age, availability, reputation and residency in the community. Candidates were presented to communities during nightly town hall meetings to receive final approval by both the traditional authorities and beneficiaries.

Beginning in October of 2016, CHWs were trained by MINSA to assume the following responsibilities: (1) Understand the biology of malaria; (2) Use RDTs and prepare thick blood smears for microscopy confirmation; (3) Apply the national *P. vivax* treatment scheme and supervise treatment; (4) Report cases using standard surveillance forms; (5) Refer children younger than 6 months, pregnant women, severe malaria cases or malaria caused by *Plasmodium falciparum* or mixed *Plasmodium* infections to the closest health facility; and (6) Promote malaria prevention, early diagnosis and treatment. The training spanned approximately three days. CHW were equipped with Standard Diagnostics Bioline Malaria P.f/Pan (HRP2/pLDH) RDTs, materials to prepare thick blood smears for microscopy confirmation, *P. vivax* treatment, and case reporting forms. The treatment regimen for *P. vivax* consisted of 3 days of chloroquine and 7 days of primaquine. CHWs were also given job aids with detailed instructions on the application of an RDT and secure waste disposal. Tests with a positive indication of pLDH only were treated as *P. vivax*. Following the pilot, CHWs were equipped with the SD Bioline Malaria Ag P.f/P.v RDT to provide species-specific detection. CHWs received a USD$50 monthly incentive for their participation.

### Data sources, indicators and analyses

#### Access to malaria care

Access to care was described over six-month intervals during the pilot using the proportion of targeted communities, and population at risk, covered by an active CHW (Table 1).

**Table 1:** Description of quantitative metrics employed to evaluate the impact of a Community Health Worker (CHW) pilot in Guna Yala, Panama

Impact parameter	Metric	Time interval
Access to Care	Percent of targeted communities with an active CHW	Six-month intervals during the pilot (October 2016–September 2018)
	Percent of the population at risk covered by an active CHW	
Quality of Care	Distribution of pre- and post-training test scores collected during trainings offered at baseline and every 6 months	Summarized by training (October 2016–September 2018)
	Distribution of supervision scores collected during routine visits made every 4 months by Vector Control Technicians	Summarized by visit number (October 2016–September 2018)
	Sensitivity: Percent of positive microscopy tests that had a corresponding positive RDT Specificity: Percent of negative microscopy tests that had a corresponding negative RDT	Summarized across the pilot period (October 2016–September 2018)
Epidemiological Impact	Blood examination rate: Number of microscopy tests/Estimated population*100 Test positivity: Number of positive microscopy tests/ Total number of microscopy tests*100 Incidence rate: Number of positive microscopy tests/ Estimated population*1000 Testing Delay: Number of days between symptom onset and test Treatment Delay: Number of days between test and treatment initiation	Study year (Y1-Y4) Pre-pilot (October 2014-September 2016) and pilot (October 2016-September 2018)

Population estimates were derived from 2010 census data published by Panama’s Instituto Nacional de Estadística y Censo [21]. Active CHWs were defined as those having tested persons seeking diagnosis for malaria and or having engaged in promotion of early testing and treatment for malaria in the community in the past four months. This was evaluated based on routine surveillance data and results of structured supervision visits made by VCTs every four months.

#### Quality of care delivered by community health workers

In addition to an introductory training, CHWs were provided with a refresher training at 6 and 12 months into their tenure. Questionnaires were administered to CHWs before and after each refresher training to evaluate knowledge gained and identify challenges that could be addressed through subsequent trainings, supervision or job aids. Tests were scored based on three key components: malaria knowledge, RDT interpretation and case management (including treatment and referrals). Tests were administered on paper and data subsequently entered into a spreadsheet for analysis. Test scores were unweighted.

During the supervision visit, VCTs evaluated CHWs’ knowledge and skills in malaria case management and diagnosis, provided feedback, managed CHW stocks, and collected test and case data. Evaluations resulted in a score that reflected knowledge of malaria symptoms and referral guidelines, and the observed use of RDTs, preparation of thick blood smears and reporting. The supervision score also incorporated responses to hypothetical case management scenarios. The score was calculated as a percent of 51 possible points, with a score of 36 (70%) required in order to pass. Supervision data were collected using SurveyCTO on tablets in the field and pushed to a GoogleSheets dashboard for routine review by VCT supervisors.

Although WHO does not recommend cross-referencing RDT and microscopy results based on biological differences in tests [22], Panama followed regional practices, comparing the results of both for quality control purposes during the pilot. In this analysis, RDTs are compared to field microscopy. Field microscopy reading was completed by a malaria-specific clinical laboratory technician within the Department of Vector Control. The technician received a 3.5-month training led by the Pan American Health Organization and participated in a quality control program led by Panama’s central reference laboratory, located at the Instituto Conmemorativo Gorgas de Estudios de la Salud.

Routine surveillance data were collected from different Excel spreadsheets maintained by the Department of Vector Control within MINSA. These included summaries of microscopy tests performed by region, month and detection strategy (2014–2018) and a distinct case registry of all microscopy-confirmed malaria cases reported in the country (2014–2018) based on a standardized case notification form. A separate CHW database, also based on information from the case notification form, was maintained in Microsoft Access and included a line listing of all persons tested by a CHW in Guna Yala during the pilot period. A combination of these three data sources was used for analyses. Information in the case registry was considered the gold standard and used to reconcile any differences between data sources.

#### Epidemiological impact of the community health worker pilot

Trends in standard surveillance and case management indicators were compared before and during the pilot. Indicators included blood examination rates, test positivity, incidence of malaria, delays between symptom onset and test date, and delays between test date and treatment initiation. Proportions were compared using chi-square tests and rates compared using incidence rate ratios (IRR). The two years prior to and two years following initiation of the CHW pilot were compared after averaging annual rates or proportions. The two-year comparison period was chosen because of the greater internal consistency of the data (e.g. consistency of place names) and additional reporting and validation conducted from 2014 onwards [23]. As a result, the study period ranged from October 2014 to September 2018 and included four years of data (Y1 = October 2014-September 2015, Y2 = October 2015-September 2016, Y3 = October 2016-September 2017, and Y4 = October 2017-September 2018). The former two years make up the pre-pilot period and the latter two encompass the pilot period. Population denominators, derived from the census data described above, corresponded to the years 2015 and 2016 (for the pre-pilot period) and 2017 and 2018 (pilot period). Surveillance indicators were based on microscopy test results and stratified by provider type. Indication of current or recent fever was used as a proxy to select for symptomatic cases in the evaluation of the delay between symptom onset and the preparation of a thick blood smear for microscopy (test date). Case management delays were compared using an Armitage test for trend. Analyses were conducted using R v.3.4.3.

## Results

### Access to malaria care

**Fig. 1:**
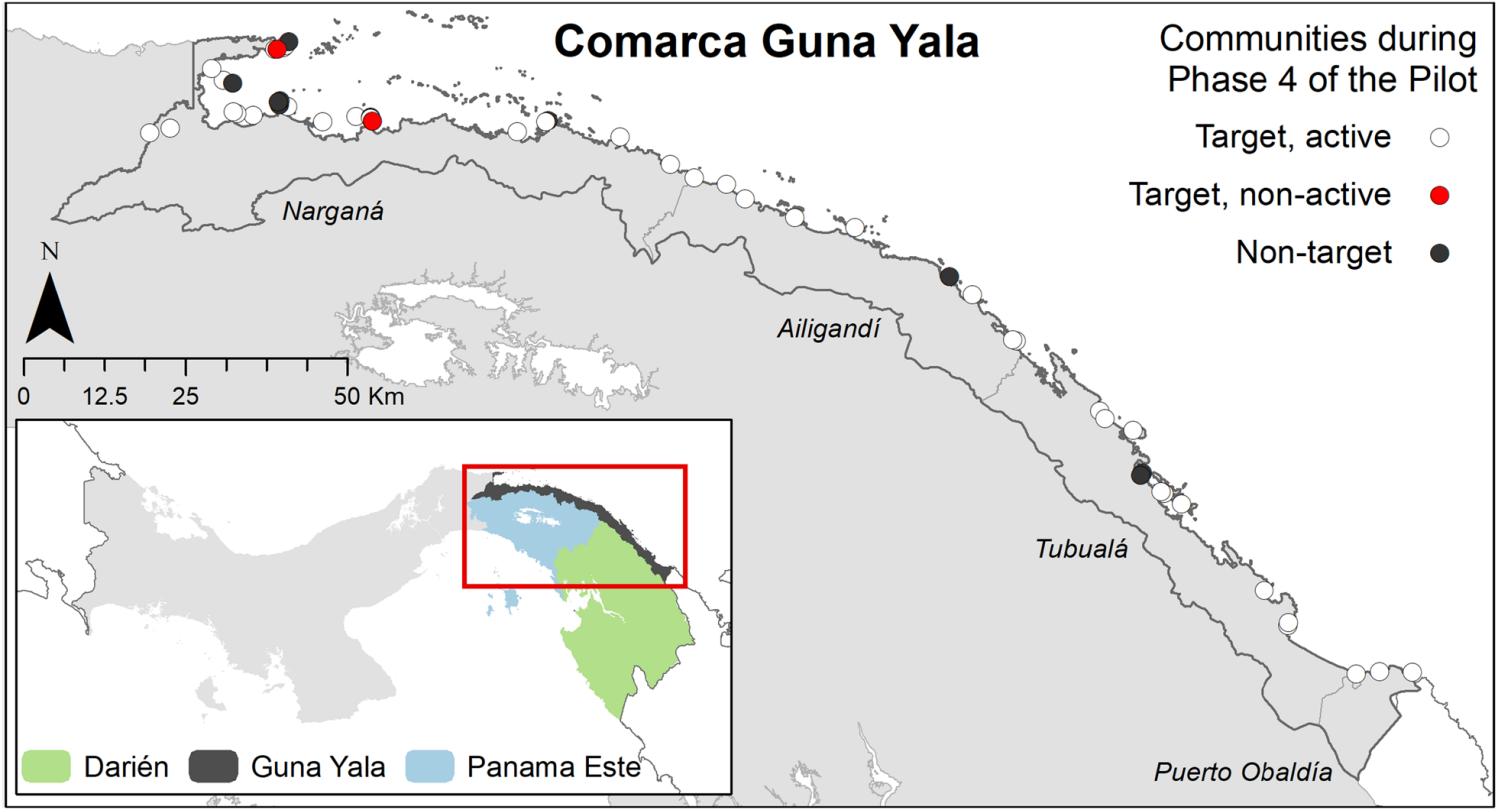
Communities in Guna Yala and status of Community Health Workers during the last six months of the pilot (April–September 2018). Most targeted communities had an active Community Health Worker in this phase, non-active communities declined to participate in the network. Inset: Map of Panama highlighting the malaria endemic health regions in the country’s east

Of the 51 communities in Guna Yala, 46 were initially targeted by the pilot programme (Table 2). During the pilot, 6 communities were eliminated from the programme because of access to an existing health facility (n = 4) or proximity to other CHWs (n = 2). One additional community was targeted because of an improved understanding of its limitations in access to care, resulting in 41 target communities. Two target communities declined to participate in the pilot citing a lack of interest. By the end of the pilot, 39 of 41 targeted communities had successfully recruited and maintained an active CHW for at least six months (Fig. 1). Reasons for CHW dropout included moving out of the community and conflicts with new employment or continued education. The network covered over 24,000 persons at risk for malaria.

**Table 2:** Targeted communities and population at risk covered by an active Community Health Worker in Guna Yala in six-month intervals during the pilot period (October 2016–September 2018)

Time Period	Target communities	Trained CHW at beginning of period	Active CHWs at end of period	Percent of target communities with an active CHW	Target population (2010 census)	Percent of target population covered by an active CHW
Oct 2016–Mar 2017	46	42	33	72%	29,671	69%
Apr 2017–Sep 2017	42	38	32	76%	25,682	79%
Oct 2017–Mar 2018	41	38	35	83%	24,656	87%
Apr 2018–Sep 2018	41	43	39	95%	24,656	97%

### Quality of care delivered by community health workers

**Fig. 2:**
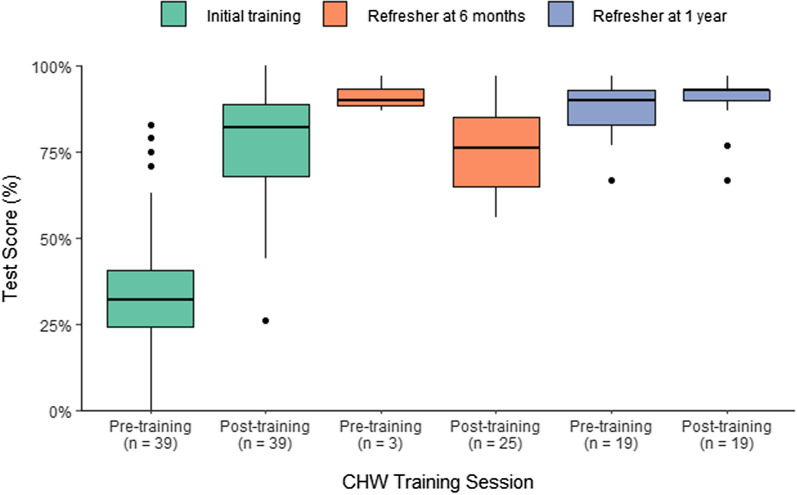
Distribution of scores for the 39 Community Health Workers active at the end of the pilot for pre-training and post-training tests during introductory and refresher training sessions (October 2016–September 2018). During one of the 6-month refresher trainings, operational constraints prevented 22 Community Health Workers from taking the pre-test

All 39 active CHWs received an initial training before beginning their work. The median test score prior to this training was 32% (range 0–83%, Fig. 2a). Following initial training, the median test score was 82% (range 26–100%). Of the 39 CHWs, 25 (64%) participated in at least one refresher training; 19 (49%) participated in two refresher trainings. Operational challenges prevented 22 CHWs from completing a pre-test during their first refresher. Median test scores were high both pre and post the one-year refresher training (90% and 93%, respectively). Results were qualitatively similar when the sample was restricted to the 19 CHWs that participated in both refresher trainings.

Following the initial training, supervision visits were made to CHWs. CHWs were visited one to eight times with an average of four visits per CHW between January 2017 and September 2018. The overall average CHW supervision score, reflecting knowledge of malaria, observation of practice, and response to hypothetical scenarios was 83% (range 60%-98%). Average scores stayed above 80% for each visit number and did not vary meaningfully over the supervision period (average score in visit 1: 85%; in visit 8: 88%).

Between October 2016 and September 2018, CHWs performed an RDT and/or facilitated a microscopy test for 1699 individuals (Table 3). Among all individuals tested, 392 individuals had a positive RDT, a positive microscopy test, or both. Both the sensitivity and specificity of an RDT compared to field microscopy in this setting were high. Among the 350 who were confirmed positive via microscopy and who had an RDT result recorded, 312 (89%) had a positive RDT. Among the 1249 individuals who tested negative via microscopy and who had an RDT result recorded, 1232 (99%) also tested negative with an RDT.

**Table 3:** Malaria tests facilitated by Community Health Workers in Guna Yala, Panama from October 2016 through September 2018

	Microscopy test result			
	Positive	Negative	NA	Total
Rapid diagnostic test result				
Positive	312	16	4	332
Negative	38	1232	54	1324
Invalid	0	1	0	1
NA	22	20	0	42
Total	372	1269	58	1699

### Epidemiological impact

#### Annual blood examination rates

**Fig. 3:**
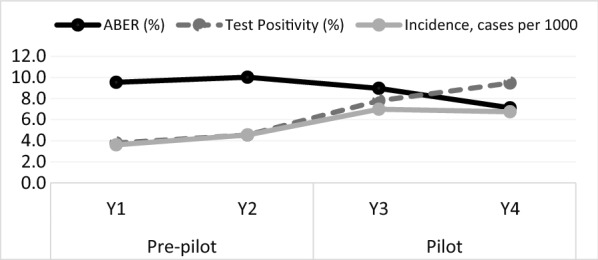
Annual blood examination rates (ABER), test positivity, and incidence of malaria in Guna Yala in the pre-pilot period (Y1, October 2014-September 2015, and Y2, October 2015-September 2016) and pilot period (Y3, October 2016-September 2017, Y4, October 2017-September 2018)

Blood smears were prepared by CHWs, health facilities and VCTs and read by a single laboratory technician in Guna Yala. The total number of microscopy tests performed across Guna Yala declined from 4043 tests in Y1 to 3220 tests in Y4, corresponding to annual blood examination rates of 9.5 per 100 persons and 7.1 per 100, respectively (Fig. 3). Significant differences were found when blood examination rates were averaged by period (pilot = 8.0 per 100 vs. pre-pilot = 9.8 per 100, IRR = 0.82, 95% CI 0.79, 0.86).

The percent of tests facilitated by VCTs through active surveillance was lower during the pilot. In the pre-pilot period, active surveillance accounted for 95.9% of all malaria tests and during the pilot, this fell to 67.8% (Table 4). Health facility testing doubled between periods (from 4.1% to 8.5%). During the pilot, CHWs facilitated nearly 24% of all tests performed.

**Table 4:** Malaria tests, confirmed cases and test positivity by surveillance strategy and provider type (CHW = community health worker, HF = health facility and VCT = vector control technician) prior to the pilot (Years 1 and Years 2) and during the pilot (Years 3 and Years 4) in Guna Yala, October 2014–September 2018

	Pre-pilot (October 2014-September 2016)	Pilot Period* (October 2016–September 2018)
**Tests Performed, (%)**	8379 (100)	7185 (100)
Passive Surveillance, CHW (%)	0 (0)	1699 (23.7)
Passive Surveillance, HF (%)	340 (4.1)	613 (8.5)
Active Surveillance, VCT (%)	8039 (95.9)	4873 (67.8)
**Confirmed Cases, (%)**	350 (100)	614 (100)
Passive Surveillance, CHW (%)	0 (0)	372 (60.6)
Passive Surveillance, HF (%)	44 (12.6)	99 (16.1)
Active Surveillance, VCT (%)	306 (87.4)	143 (23.3)
**Test Positivity, (%)**	4.2	8.5
Passive Surveillance, CHW	–	21.9
Passive Surveillance, HF	12.9	16.2
Active Surveillance, VCT	3.8	2.9

^*^Four cases in the pilot period were missing a provider type and were excluded from the table

#### Test positivity

Test positivity more than doubled from Y1 (3.8%) to Y4 (9.5%), with the largest increase taking place once the pilot once was fully implemented in Y3. Test positivity significantly increased between the pre-pilot and pilot periods (pre-pilot = 4.2%, pilot = 8.5%, Χ^2^ = 126.3, df = 1, *p* < 0.001). Trends in test positivity varied by provider, increasing among cases presenting to health facilities (from 13 to 16%) and decreasing among those found by VCTs between pre- and pilot periods (from 3.8% to 2.9%). During the pilot, test positivity was highest in those tested by CHWs (21.9%).

#### Confirmed cases and incidence

Between October 2014 and September 2018, there were 968 cases reported by the region of Guna Yala. Approximately 58% of cases were male and 12% were younger than 5 years. Between the pre- and pilot period, the number of reported cases increased from 350 to 618. Reported incidence increased from an average of 4.1 cases per 1000 to 6.9 cases per 1000 (IRR = 1.83, 95% CI 1.52, 2.21). During the pilot, CHWs detected approximately 61% of all reported cases while VCTs detected approximately 23% and health facilities, 16%.

Reported cases were all caused by *P. vivax* with the exception of three *P. falciparum* cases imported from Colombia during the pre-pilot period, and possibly also, 4 locally acquired cases with missing species information in Y4. During the study, 54 imported cases were reported; 50 of which were from Colombia, 3 from Cuba and 1 from Italy, reflecting a possible reporting or classification error as Italy does not have endemic malaria. There were 19 imported cases in the pre-pilot period and 35 in the pilot period, making up 5.4% and 5.6% of all reported cases, respectively. VCTs detected 17 (89%) of the imported cases before the introduction of CHW after which, they only detected 2 (6%). CHWs detected 27 (77%) of all imported cases in the pilot period; 23 of these cases were detected in Puerto Obaldía, the corregimiento closest to the Colombian border.

#### Case management delays

**Fig. 4:**
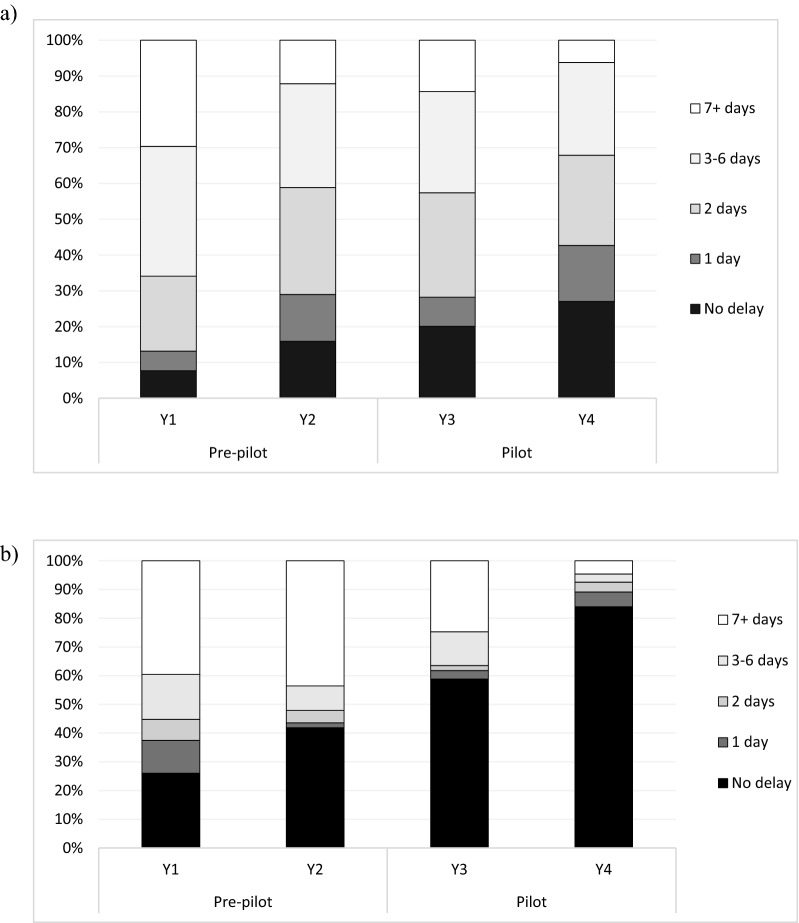
Malaria case management delays in Guna Yala in the pre-pilot period (Y1, October 2014-September 2015, and Y2, October 2015-September 2016) and pilot period (Y3, October 2016-September 2017, Y4, October 2017-September 2018). **a** Diagnostic delays are represented by the number of days between symptom onset and test date and **b** Treatment delays (N = 681) as the number of days between test date and initiation of treatment (N = 558)

Among the 968 cases, 920 (95%) reported being recently or currently febrile at the time of thick blood smear preparation for a microscopy test (test date). Among febrile cases, 222 were missing a date of symptom onset and 17 reported symptom onset after their test date, leaving 681 cases with record of symptom onset prior to the test date. The percent tested on the day of symptom onset increased each year (Y1 = 8%, Y2 = 16%, Y3 = 20%, and Y4 = 27%, Fig. 4a). There was strong evidence for a trend towards reduced diagnostic delays during the pilot compared to the pre-pilot period (z = 4.8, *p* < 0.001).

Among the 968 cases detected, 389 were missing a date of treatment initiation and 21 cases began treatment before their test date. The proportion of cases missing dates of treatment initiation were roughly equally distributed across study years (range = 35%-43%). Among the remaining 558 cases, 321 (58%) initiated treatment on the day of their test. The proportion treated on the day of their test increased each year (Y1 = 26%, Y2 = 42%, Y3 = 59%, Y4 = 84%, Fig. 4b). There was a significant trend towards reduced treatment delays during the pilot compared to the pre-pilot period (z = 8.6, *p* < 0.001).

## Discussion

The CHW network improved access to malaria diagnosis and treatment in a high-incidence and remote region of Panama. The introduction of CHWs in Guna Yala successfully extended care to 39 communities, reaching over 24,000 persons at risk for malaria. Consistently high supervision visit scores, as well as high sensitivity and specificity of RDT results compared to field microscopy, lend evidence to CHWs’ proficiency in utilizing RDTs in the field and the reliability of RDTs as a diagnostic tool in this setting. A proficient network of CHWs equipped with high-quality RDTs allows public health entities, like MINSA, to be more confident in their surveillance data and the safety of withholding treatment to test-negative patients (22). The introduction of RDTs during the pilot led to a policy change at the national level in 2018, which allowed for malaria confirmation via RDT. Based on results from the pilot, the CHW network was subsequently scaled up to all other malaria endemic regions in the country (Darién, Ngäbe-Buglé and Panamá Este). As of 2022, the network in Guna Yala remains active and is reviewed on a yearly basis to determine if changes in epidemiology or access to care merit the removal or addition of target localities.

Case management provided by CHWs allowed VCTs to repurpose their time. During the pilot, the number of tests conducted by VCTs was nearly cut in half. VCTs’ duties shifted to include supervision of CHWs, validation of data collected on reporting forms, data review and more vector control. The increased number of tests conducted by health facilities during the pilot may have been the result of CHW referrals, strengthening passive surveillance overall. With new data coming in from passive surveillance efforts, as well as other surveillance system improvements including rapid reporting and data visualization, VCTs can better design, target and respond with, specific interventions.

The introduction of CHWs in Guna Yala led to an increase in the number of reported cases. The increase cannot be attributed to higher testing rates, as has been observed in other countries scaling up access to diagnosis [24]. Test positivity resulting from CHW tests was five times higher than among tests conducted by VCTs prior to the pilot, suggesting that CHWs targeted high-risk populations without prior access to malaria diagnosis and testing. Before the introduction of CHWs, testing quotas applied to VCTs may have resulted in a test and treat strategy on the ground instead of the documented protocol, which was to screen, test and treat. The deviation in protocol would have led to less efficient case detection.

It is also possible that the higher test positivity and incidence during the pilot reflected a true increase in transmission. Natural increases in malaria transmission in Panama have been linked to the El Niño Southern Oscillation [3, 8, 25], which was particularly hot and dry during the years 2015–2016 [3]. However, test positivity among tests conducted by VCTs decreased between the pre-pilot and pilot period. Published data from the neighbouring region of Darien indicate a relatively stable test positivity and incidence during the study period [26], which would refute the assertion that there was an underlying increase in transmission.

The high-test positivity among tests performed by CHWs is an indicator that more testing may be warranted in specific communities. In these areas, the CHW network could be supported to raise awareness about malaria and to promote testing at the community level. In other malaria elimination contexts, the integration of other health services such as treatment for childhood diarrhea and pneumonia, has been shown to increase demand for CHW services [27]. Broader levels of support could be provided to CHW, such as integration with health department staff working in other disease areas and local health clinics, in order to expand the package of services provided by CHWs [28].

The proportion of cases that were detected and treated earlier significantly increased following the introduction of CHWs. Yet, in the final year of study, over half of all cases waited two days or longer following their first symptom to seek care. This analysis indicates a need to further generate demand for CHW services and encourage prompt care seeking. The predominance of *P. vivax* infection in Panama results in additional challenges related to treatment of the parasite. A 14-day primaquine treatment (or, 7-day regimen applied in remote regions like Guna Yala) is needed for radical cure. Not only will the lengthy treatment require more time from CHWs because it is observed, but it will require that CHWs motivate cases to complete treatment even after symptoms have subsided. Additional efforts underway to support the network to provide radical cure include updating CHW trainings to include specific sessions on the importance of treatment adherence, development of promotional campaigns focused on treatment completion, closer supervision of CHWs and patient follow-up by VCTs and local health facilities.

The limited resolution, availability and quality of the data prevented a more comprehensive evaluation of the CHW pilot. For example, the number of tests performed were unavailable at community level throughout the study period. Thus, surveillance indicators were aggregated across all communities in Guna Yala. Additionally, reports of treatment were largely missing and not available electronically which prevented the analysis of treatment completion. A substantial proportion of cases were missing date of symptom onset and date of treatment initiation which introduces considerable uncertainty in the case management timelines. Furthermore, date of symptom onset is self-reported and the reliability of this measure may be low. Qualitative information regarding CHW motivation, alignment of the CHW incentive to their workload, and community perception of the health services offered were not evaluated but could be important to address the success of this pilot.

Panama remains committed to malaria elimination and in line with its commitment to the 2030 Agenda for Sustainable Development, whose third goal calls for the good health and well-being of populations [29], it will be important to maintain uptake of CHW services and health system support to the CHW network. These may require network institutionalization and integration of other health services provided by CHWs to ensure service relevance, sustained financing in the years ahead and CHW motivation. The network’s contributions in Guna Yala highlight a significant opportunity to expand community-based service provision to other diseases, to bridge barriers in health service access and uptake and to address historic health inequities in Panama’s indigenous regions.

## Data Availability

Aggregate and anonymized data are available from the corresponding author upon reasonable request and with permission from the Ministerio de Salud de la República de Panamá.
